# Exosomal miRNAs as biomarkers in predicting chemotherapy-induced cardiotoxicity in patients with cancer (Review)

**DOI:** 10.3892/mi.2025.268

**Published:** 2025-09-04

**Authors:** Vedant Shah, Viraj Panchal, Abhi Shah, Bhavya Vyas, Subham Bhowmik, Ishita Panchal, Pragya Jain

**Affiliations:** 1 Department of Internal Medicine, The New York Medical College Graduate Medical Education Program at St. Mary's General Hospital and Saint Clare's Health, Denville, NJ 07834, USA; 2 Department of Medicine, Smt. N.H.L. Municipal Medical College and SVPISMR, Ahmedabad, Gujarat 380058, India; 3 Department of Family Medicine, Hennepin Healthcare, Minneapolis, MN 55415, USA; 4 Department of Neurology, Max Smart Hospital, New Delhi 110017, India; 5 Department of Medicine, Jawaharlal Nehru Medical College, Belagavi, Karnataka 590010, India; 6 Department of Internal Medicine, Baptist Hospital of Southeast Texas, Beaumont, TX 77701, USA

**Keywords:** exosomal microRNAs, cardiotoxicity, chemotherapy-induced cardiotoxicity

## Abstract

With the advancements made in oncology, such as molecularly targeted therapies and immune checkpoint inhibitors, their use has significantly improved the outcomes of patients with cancer. In addition, these advancements have led to an increased incidence of cardiotoxicity, ranging from subclinical dysfunction to severe cardiomyopathy. Hence, it is imperative to enable the early detection and prevention of cardiotoxicity to mitigate irreversible cardiac damage. Exosomal microRNAs (miRNAs) have emerged as promising non-invasive biomarkers due to their stability in biological fluids and their ability to reflect early myocardial stress and damage. Unlike traditional biomarkers such as troponins, which indicate damage after myocardial injury has occurred, miRNAs can detect subclinical changes earlier, enabling timely intervention. Exosomal miRNAs not only serve as diagnostic biomarkers, but also provide therapeutic potential by modulating molecular pathways associated with cardiotoxicity. Understanding the regulatory mechanisms of miRNA expression in chemotherapy-induced cardiotoxicity can pave the way for personalized cardioprotective strategies, minimizing cardiovascular complications during cancer therapy. The present review discusses the role of various miRNAs as biomarkers for the early diagnosis of cardiotoxicity induced by chemotherapeutic agents.

## 1. Introduction

Over the years, chemotherapy has made significant progress in the treatment of various oncological conditions. However, along with these advancements, several side-effects have emerged. One of the most critical is cardiotoxicity, which can lead to heart failure, arrhythmias and other cardiovascular complications (1). However, the extent of cardiotoxicity is dependent on various factors, including the type of chemotherapeutic agent used, the dose used and pre-existing cardiac conditions (2).

Patients experiencing these side-effects may experience difficulty with day-to-day activities, which may require modifications to the regimen, potentially compromising the efficacy of the treatment and its outcome. Early detection aids in timely intervention and can prevent the progression of cardiac damage, hence becoming crucial in managing chemotherapy-induced cardiotoxicity.

Liquid biopsy is a term used to describe the testing of bodily fluids; however, in the cancer diagnostic field, it focuses on tests that target a specific biomarker. It is non-invasive and cost-effective and can also be used to monitor the cardioprotective strategies used during chemotherapy regimens (3). Exosomal microRNAs (miRNAs/miRs) can be used as one of the targeted biomarkers that provide insight to the molecular pathology involved in myocardial damage. miRNAs are short, single-stranded RNA sequences consisting of 18 to 24 nucleotides in length. They are non-coding RNA molecules and play a crucial role in the regulation of gene expression. They are usually abundantly present in the cytoplasm but some of them can also be released into the blood. These circulating miRNAs are potentially a good prospect as a diagnostic biomarker for cardiotoxicity as they are resistant to degradation and provide direct information on the mechanism of injury (4). The present review provides discusses the efficacy of certain promising tools, such as exosomal miRNAs, which can be used to predict chemotherapy-induced cardiotoxicity.

## 2. Chemotherapy-induced cardiotoxicity: Mechanisms and clinical impact

With the advancements that are ongoing in cancer therapies, the risk of developing associated adverse events is increasing. Among these, cardiovascular adverse events of anticancer agents have been reflected in the field of cardio-oncology. These events are commonly associated with heart failure with reduced ejection fraction, arrhythmias, thrombosis, hypertension, ischemia/myocardial infarction and a range of other potential cardiovascular conditions (5). The majority of the effects cause irreversible cardiac damage with increased morbidity and mortality among the patients receiving the chemotherapy (6). With the continuous ongoing research, it is crucial to elucidate the underlying mechanisms of cardiotoxicity associated with anticancer therapy for early detection and management the patients who are at risk.

There are several agents associated with cardiotoxicity, such as anthracyclines, alkylating agents, HER-2 targeted therapies, antimicrotubule agents and antimetabolites (7). Among these, the most commonly associated are anthracycline agents with a rate of 0.2-8.7%, which increases with cumulative dosing (8). These agents include doxorubicin (DOXO), daunorubicin, epirubicin and idarubicin used in the treatment of lymphomas, leukemias, breast cancer and sarcomas (9,10). Their cardiotoxic effects can lead to left ventricular ejection fraction (LVEF) dysfunction, potentially progressing to heart failure, as well as cardiac arrhythmias (10).

**Figure 1:**
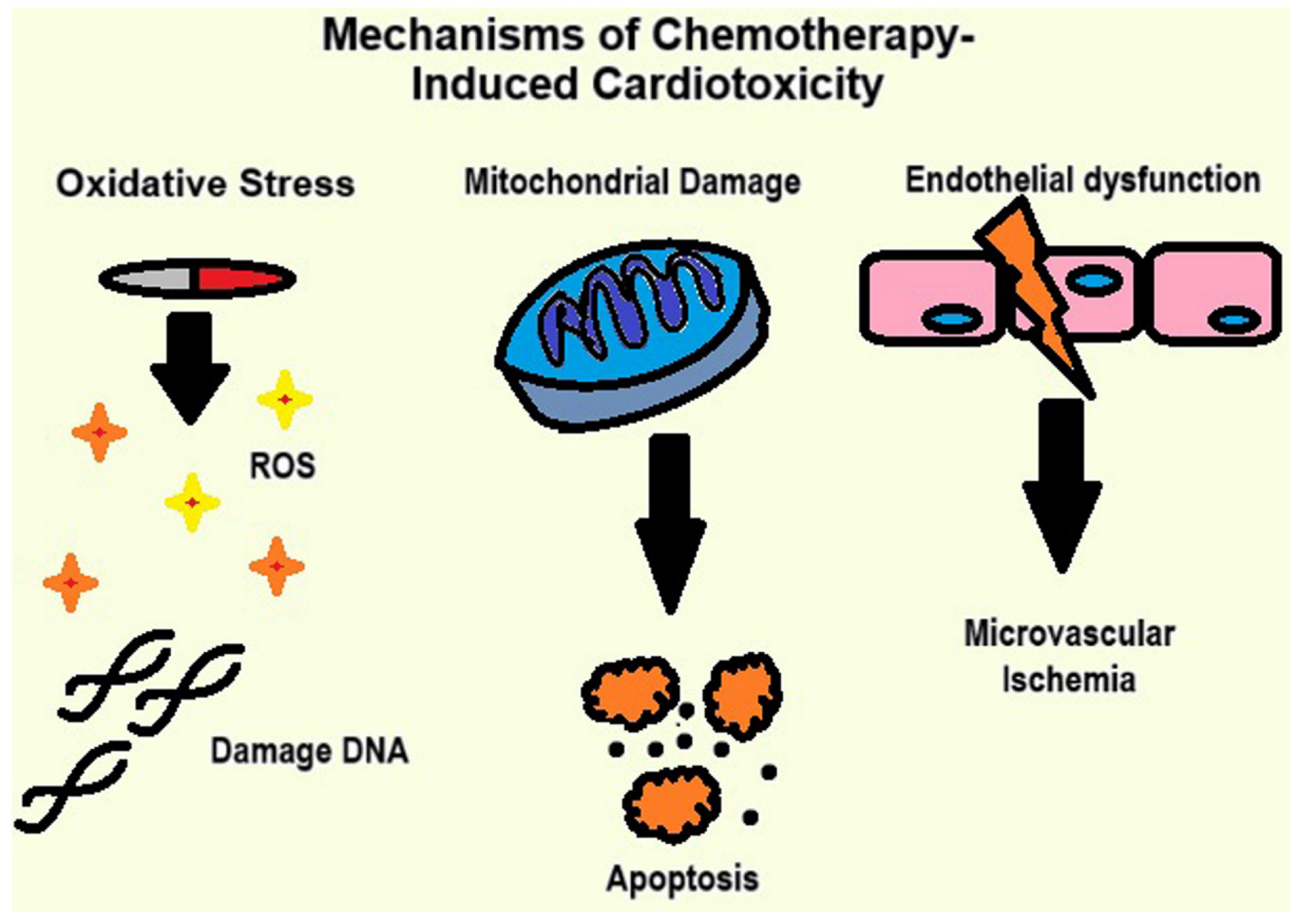
Image demonstrating the key mechanisms of chemotherapy-induced cardiotoxicity.

The mechanisms behind the damage range from oxidative stress to mitochondrial damage, and vascular injury, as illustrated in Fig. 1. The key mechanisms of chemotherapy-induced cardiotoxicity (CIC) include the following: i) Oxidative stress: Chemotherapeutic agents, such as anthracyclines generate reactive oxygen species (ROS), leading to damage to cellular proteins, lipids and DNA (11); ii) mitochondrial damage: ROS accumulation impairs mitochondrial function, disrupts ATP production and triggers cardiomyocyte apoptosis (12), and iii) endothelial dysfunction: Damage to endothelial cells disrupts vascular homeostasis, reduces nitric oxide availability and promotes coronary microvascular ischemia (11).

CIC affects the efficacy of the treatment and the quality of life of patients. The treatment may be compromised when the occurrence of CIC causes a reduction in dosage, or an early end to pause the chemotherapy regimen due to associated damage (13,14). Due to the irreversible damage caused by CIC, it becomes essential to detect the events.

Currently, cardiac monitoring in patients with cancer relies on the use of echocardiography and serum biomarkers, such as troponins or natriuretic peptides detected after damage has occurred (15). There is a need for highly sensitive and specific biomarkers to identify early or subclinical cardiotoxicity prior to the occurrence of damage. Newer research focuses on the use of molecular markers such as miRNAs, and circulating endothelial cells which can predict the risk of cardiotoxicity at an earlier stage (16). There is an unmet need for early predictive marker detection and integrating those biomarkers in standardized guidelines to provide routine oncology care. The use of miRNAs as one of the molecular markers can significantly reduce the burden of CIC and enhance patient outcomes.

## 3. Exosomes and their role in intercellular communications

One of the critical components and regulators of the tumor environment are exosomes. They are lipid bi-layer vesicles ranging from 30 to 150 nm in diameter, which are often released from the cells and resemble the lipid bilayer of the parent cell (17). They are formed by the inwards budding of the plasma membrane of particular late endosomes to form multivesicular bodies which are released outside the cell membrane (18). Such exosomes participate in intercellular communications, and cell-to-cell communications to regulate diverse signaling pathways or, present the B-lymphocyte antigens marking their important role in immune-related functions (19,20).

There are various mechanisms through which exosomes communicate with the recipient cells, such as by binding to the surface of the recipient cell through adhesion molecules on exosomes, fusing after the adhesion with the membrane, through receptor-mediated endocytosis, or by phagocytosis (21). Through such an interaction, there is an exchange of the contents from the exosomes into the recipient cells such as growth factors, or transfer of genetic material (21). The transfer of molecules such as protein, RNA, and DNA causes the regulation of pathways of recipient cell at particular sites. The presence of both miRNAs and mRNAs within exosomes and the transfer of these contents is responsible for cell-to-cell communications (21). Previous research has demonstrated the presence of several non-coding RNA species such as miRNAs, transfer RNAs, or small non-coding RN within exosomes; these are associated with the function of various genes involved in cellular regulation (22). In the early 21st century, it was found that exosomes also contained various RNA species, such as miRNAs and mRNAs, which participate in intercellular communications and are responsible for multiple pathophysiological processes in tumor suppression or progression, tumor immunity, or inflammation (17,23-25). It was later found that exosomes are secreted in large quantities by tumor cells with protein and RNA species responsible for tumor progression (26,27). They are primarily involved in intercellular communications between the tumor and normal cells by affecting the MAPK/ERK signaling and miR-338/MACC1/MET pathways promoting tumor growth and invasion (28,29). Various exosomal families have been found in a proteomic study of colorectal cancer which exhibited enriched KRAS, EGFR, and SRC family kinases in exosomes (30). Of note, breast cancer-associated exosomes have been found to contain pre-miRNAs and the RNA-induced silencing complex-related proteins, Dicer, AGO2 and TRBP, which are crucial during miRNA biogenesis (31). Additionally, the amount of miRNA released per exosome differs between various cells and tissues. Although the quantity of miRNA obtained may be low, it is highly specific to the target cells and carries out distinct functions (32,33).

The presence of miRNAs and mRNAs inside exosomes of B16F0 tumor cells was also found in a previous study (34). Of note, another study demonstrated that the miRNA contents of tumor-derived exosomes differed significantly from those of normal cells, with a unique set of miRNAs involved in epithelial cell differentiation (35). Additionally, various studies have shown that exosomes with multiple miRNAs could promote a pre-metastatic niche or upregulate a distinct set of genes responsible for tumor cell progression. Xu *et al* (36) demonstrated that lung adenocarcinoma cell-derived exosomal miR-21 promoted osteoclastogenesis and was associated with osteolytic metastasis. Similarly, Yang *et al* (37) demonstrated that exosomal miR-423-5p was responsible for the growth and metastasis by repressing SUFU protein expression and in turn, promoting the cellular proliferation and spread of recipient gastric cells. Furthermore, miR-675 has been found to be responsible for cellular migration and invasion by upregulating CALN1 protein expression, and exosome miR-103 can promote metastasis by directly targeting junction proteins, such as VE-Cadherin, p120-catenin, and zonula occludens; miR23a secreted from nasopharyngeal carcinoma can promote angiogenesis by the repression of the *Tsga10* gene (38,39).

With the advent of the role of exosomes in cellular communication, it should be noted that they have been used as ‘fingerprints’ or ‘signatures’ for the cell of origin, particularly with miRNAs, proteins, or lipids that mimic the cellular origin (40-43). High levels of serum exosomal miR-21 have been significantly linked with esophageal squamous cell cancer which helped to distinguish those patients from patients who were diagnosed with benign diseases (44). Furthermore, patients with hepatocellular carcinoma were also noted to have higher levels of serum miR-21 compared with chronic hepatitis B or healthy volunteers (45). Various exosomes have been detected to be linked with increased diagnostic values, some of which are described in Table I.

However, the exact role of these exosomal contents (proteins or miRNAs) as biomarkers has not yet been explored in clinical practice. With the increasing awareness and readiness to obtain fluids rich in exosomes, one can use specific miRNAs of the exosomes in clinical diagnosis as well as monitor the toxicity of a therapeutic agent.

## 4. Exosomal miRNAs in cardiotoxicity: Evidence from preclinical and clinical studies

Currently, with the recent advancements being made in oncology with the use of molecular targeted therapy and the use of immune checkpoint inhibitors, the frequency and severity of cardiotoxicity, such as subclinical ventricular dysfunction to severe cardiomyopathy remains elevated (46-48). Hence, the early detection and prevention of cardiotoxicity before it progresses to an irreversible stage is of utmost importance. The roles of miRNAs functioning as a key regulators in cellular proliferation, cell death and apoptosis have been elucidated. However, the dysregulation of miRNAs has been shown to be associated with adverse cardiac remodeling and toxicity, which can provide therapeutic potential, particularly in diagnoses and in understanding the cardiotoxicity induced by various chemotherapies (49). There are several miRNA families whose profiles have been linked to the cardiotoxicity secondary to chemotherapy; these are described below with the specific mechanisms responsible for the regulation of the particular miRNA and are also listed in Table II.

### miRNA-200 family

This family of miRNAs is involved in epithelial-to-mesenchymal transition, where their expression can be modulated due to the chemotherapeutic agents (50). One of the most common agents that has been linked to the induction of miRNA-200c is DOXO. DOXO induces the upregulation of miRNA-200c expression in cardiac mesenchymal progenitor cells (51). This occurs as miRNA-200c is an oxidative stress-induced miRNA, which when upregulated, leads to endothelial dysfunction and its expression increases with DOXO in cardiomyocytes (52). The increased expression of miRNA-200c reduces the production of the ZEB1 protein, which in is turn associated with the induction of apoptosis and senescence in endothelial cells. Apart from this, the decreased expression of ZEB1 protein leads to senescence in endothelial cells (53). It has also been found that miRNA-200c is associated with decreased endothelial nitric oxide production and the downregulation of sirtuin 1 (SIRT1) and forkhead box O1, both of which play critical roles in maintaining endothelial cell homeostasis (54).

Another miRNA that is affected by DOXO is miRNA-200a, which is involved in lowering oxidative stress, since it targets Kelch-like ECH-associated protein 1, which results in the activation of nuclear factor erythroid 2-related factor 2(55). Notably, DOXO administration results in lower levels of miRNA-200a, causing the loss of the protective effect against increased oxidative stress and resulting in cardiac apoptosis, without markedly affecting matrix metalloproteinase and inflammatory factors in mice (55).

The cardiotoxicity caused by the DOXO-induced upregulation of miRNA-200c can be partially reverted by the administration of stromal cell-derived factor 1 (SDF1), which was previously found in a mouse model (51). There was an interplay between the ZEB1 mRNA, which is induced by DOXO, and p53 which is inhibited by ZEB1 protein (51). Thus, by injecting SDF1 in DOXO-treated mice, it was found that the cardiotoxicity was redacted and also partially reverted the remodeling effect, decreased left ventricular end-diastolic volume, ejection fraction, and the recovery of left ventricular end-systolic pressure (51).

### miR-34 family

The miR-34 family contains three homologous miRNAs, namely miRNA-34a, b and c; these miRNAs play a major role in anthracycline-induced cardiotoxicity (56-58).

There are various mechanisms through which miRNA-34a upregulation is responsible for the cardiotoxicity mediated by DOX in rats (58). The levels of miR-34a have been found to be increased in the plasma of patients with diffuse large B-cell lymphoma treated with epirubicin (58). One of these is through the induction of BCL2-associated X, apoptosis regulator (Bax), which inhibits B-cell lymphoma 2 (Bcl-2) expression, activating caspase-3 and mitochondrial potentials. Additionally, miR-34a results in the deacetylation of the p66hcA gene promoter by targeting SIRT1(59). This leads to an enhanced p66shc protein expression, leading to increased ROS generation in mitochondria and generating oxidative signals (60). Thus, miR-34a affects oxidative stress and contributes to the cardiotoxicity of DOX by regulating the SIRT1/p66shc pathway (58). This was also observed when the levels of miR-34a were increased in the myocardium and plasma of DOX-treated rats and cardiomyocyte H92c cells treated with DOX (58). However, dexrazoxane primarily used to prevent anthracycline-induced cardiomyopathy can reverse the increased levels of miR-34a levels (58).

Apart from its role in anthracycline-induced cardiotoxicity, miR-34a has also been shown to contribute to radiation-induced cardiotoxicity in human myocytes, primarily through regulation by the p53 oncoprotein, which is activated in response to ionizing radiation (61,62). As previously demonstrated, miR-34a plays differential roles in radiation exposure, influencing both tumor radioresistance, defined as the ability of tumor cells to survive and adapt to radiation therapy and tissue radiotoxicity, leading to collateral damage inflicted to surrounding healthy tissue (62). When ionizing radiation was used on human cardiomyocytes, the migration inhibitory factor, a cardio-protective cytokine, was shown to decrease miR-34a levels, resulting in reduced radiation-induced damage by upregulating SIRT1(62).

Additionally, miR-34a is an immunotherapeutic agent as it targets PD-L1. which is the primary target of several immune checkpoint inhibitors. In mice, autoimmune myocarditis and PD-1 deletion due to PD-L1 suppression can result in dilated cardiomyopathy, decreased contraction and heart failure (63-66). Thus, the modulation of miR-34a has been explored in a liposomal form which can be used as an immunotherapy strategy (67).

Furthermore, in the miR-34 family, miR-34b/c has also been found to be upregulated in the DOX-treated murine adult cardiomyocyte cell line, HL-1(56). It was found that itchy E3 ubiquitin-protein ligase (ITCH) was the direct target of miR-34b/c, which caused decreased HL-1 viability, eventually promoting NF-κB expression and increasing tge levels of proinflammatory cytokines such as TNF-α and IL-6 through ITCH downmodulation (56).

Notably, miR-34a appears to play a dual role, contributing to both cardiotoxic and cardioprotective mechanisms depending on the biological context. For example, while miR-34a upregulation has been linked to radiation-induced cardiotoxicity via the p53/SIRT1/p66Shc axis (58,62), it also exerts immunomodulatory effects by targeting PD-L1, which may play a protective role in autoimmune cardiomyopathy (63,66). These context-dependent effects may be influenced by factors, such as the differential expression of miR-34a targets in various cell types, the availability of RNA-binding proteins, or post-transcriptional modifications (e.g., 3' end trimming, uridylation, or adenylation) that affect miRNA stability, localization and binding specificity (68,69). Additionally, the tumor microenvironment, oxidative stress levels and the co-expression of competing endogenous RNAs may further modulate miR-34a activity (12,70). Understanding these regulatory layers could help clarify the mechanisms through which the same miRNA can mediate divergent effects and guide therapeutic strategies targeting miR-34a in a context-specific manner.

### miR-29 family

The miR-29 family consists of mi2-29a, b and c, which affect the apoptotic pathways. They are mainly responsible for the inhibition of cardiac fibrosis and affect the cardiac remodeling during the cardiac injury (71). As previously demonstrated, miR-29a levels were upregulated following myocardial injury and were directly correlated with the extent of the late remodeling, whereas significantly high levels of miR-29a levels were noted in the plasma of patients who had cardiac hypertrophy and lower levels in those who reached cardiac fibrosis (72,73).

Another member of the miR-29 family, namely miR-29b directly targets Bax, an anti-apoptotic protein, and prevents cardiac myocyte damage due to DOX therapy (74). It was previously demonstrated that miR-29b expression was downregulated in the myocardium of DOX-treated rats (74). Eventually, when miR-29b was overexpressed, it resulted in the rescue of DOX-mediated cardiotoxicity (74). This was also supported in another study when the post-anthracycline therapy levels of miR-29b were elevated in the plasma of patients in a dose-response manner with the anthracycline dose and played a role in indicating a marker of cardiac injury (75).

One of the reasons behind this is that miR-29b affects the genes involved in the extracellular matrix (ECM), such as fibronectin, collagen and matrix metalloproteinases (76). When an anthracycline-induced cardiac injury occurs, it affects the early and late ECM remodeling and hence, the upregulation of miR-29 occurs as a response to the anthracycline-induced injury (77,78).

Apart from anthracyclines, miR-29b levels are downregulated in irradiated vs. non-irradiated arteries as miR-29b particularly targets pentraxin-3 and dipeptidyl-peptidase 4, which play a role in regulating the inflammatory and matrix protein binding (79). It has been observed that lower levels of miR-29b can significantly increase the vascular inflammatory response (79). Additionally, plasma levels of miR-29a have been found to decrease in patients with non-small cell lung cancer (NSCLC) following radical thoracic radiotherapy; this is associated with the radiotherapy dose administered (80). These findings suggest a potential link between the downregulation of miR-29 and increased vascular inflammation following radiotherapy. However, further studies are required to validate whether miR-29 family members can serve as reliable predictive biomarkers in this context.

### miR-30 family

Consisting of five members from miR-30a, b, c, d, and e, this family plays a role in cardioprotection. When expressed, it decreases the contractile response to β-adrenoreceptors in cardiomyocytes. Its expression is decreased following DOX treatment and induces a decrease in the viability of cardiomyocytes (81). DOX affects GATA6, a transcription factor in cardiac development that inhibits miR-30 transcription, thus resulting in the downregulation of miR-30 following DOX treatment (81). A previous study demonstrated that in patients who were receiving bevacizumab for NSCLC, apart from anthracycline-induced damage, the levels of miR-30 decreased and were associated with cardiotoxicity during chemotherapy (82). This indicated that the levels of miR-30 may be used to assess the cardiotoxicity pre- and post-chemotherapy to monitor the cardiotoxicity (82).

### miR-21

miR-21 plays both a negative and protective role in cardiotoxicity. In a previous study, when DOX was used chronically in rats, the levels of miR-21 were found to be upregulated in cardiomyocytes, whereas acute treatment with DOX did not markedly alter these levels (83). Similarly, the levels of miR-21 increased in H9C2 cells when exposed to DOX at various concentrations (83). There are several mechanisms through which miR-21 exerts its anti-apoptotic functions. One of these is its role in ischemia-induced cardiomyocyte death, which is mediated through the direct inhibition of targets, such as programmed cell death 4 and activator protein-1, which are pro-apoptotic factors eventually leading to the induction of cardioprotective mediators such as endothelial nitric oxide synthase, heat shock protein 70 and heat shock transcription factor-1 (84,85). Another mechanism involves the inhibition of B cell translocation gene 2, which is involved in the proliferation, DNA damage repair, differentiation and apoptosis of cancer cells (83).

Apart from this, miR-21 has also been shown to affect fibrosis and remodeling by targeting the expression of PTEN (86). Lastly, miR-21 affects the survival of fibroblasts and growth factor secretion to control interstitial fibrosis. It has been found that the miR-21 levels are elevated in cardiac fibroblasts in mice and its knockdown is able to regress the cardiac fibrosis and hypertrophy in mice (87).

Additionally, the upregulation of miR-21 occurs in radiation-induced toxicity, modulating extracellular matrix proteins and PKC signaling, which affect the electrical coupling of connexin 43(88). miR-21 levels were also detected following acute genitourinary radiotoxicity in peripheral mononuclear cells (89).

The depletion of miR-21 also plays a pivotal role in immune regulation, involving the expression of PD-L1 and activating STAT1 in cultured bone marrow-derived macrophages and tumor-associated macrophages residing in tumors. Thus, PD-1 antibodies and miR-21 deficiency act synergistically as antitumor therapy (90). Strategies to inhibit miR-21 could decrease the cardiotoxicity-related events associated with chemotherapy.

### MyomiRs

There is a family of miRNAs with a specific role in the survival, proliferation and differentiation of muscles; hence, these are studied for their role in cardiac homeostasis and cardiotoxicity induced by several chemotherapeutic agents (91).

Among these, miR-1 is a skeletal muscle-specific miRNA that is involved in the differentiation of cardiomyocytes and exerts an anti-proliferative effect (92,93). It has been found that miR-1 is upregulated in the rat myocardium during ischemia/reperfusion injury, as well as in patients suffering from myocardial injury (94,95). Another study further demonstrated that the serum levels of miR-1 were associated with the size of the infarct and the levels of creatine kinase-myocardial band (96). Notably, the miR-1 levels were associated with changes in LVEF to a greater extent than the cTnl levels (97).

miR-133 is another myomiR involved in cardiac hypertrophy and associated with cardiac hypertrophy (85). Additionally, miR-133a/b has been demonstrated to exhibit anti-apoptotic properties by the inhibition of caspase-9 expression (98). Another study also demonstrated that when DOX-mediated cardiotoxicity was monitored, the levels of miR-133a/b were increased in plasma (99).

miR-208a/b is involved in the regulation of myosin heavy chain isoform switch occurring during the development and pathophysiology. As previously demonstrated, in DOX-treated mice, there were increased levels of miR-208a in hearts, which led to cardiomyocyte apoptosis. Hence, the downregulation of miR-208a could counteract myocyte apoptosis in DOX-treated animals and can be used as a plasma biomarker for cardiotoxicity in rats (97,100). Characteristically, miR-208b is exclusive to healthy human hearts and studies have been ongoing to elucidate its role (101).

Lastly, miR-499 is a myomiR whose plasma levels in children and young adults are significantly and positively associated with the anthracycline dose (75). In addition, it was previously demonstrated that miR-499 was downregulated in the hearts of DOX-treated mice, as miR-499 targets p21. The downregulation of p21 is responsible for mitochondrial fission and cell death of myocytes exposed to DOX, while miR-499 expression in serum was increased (102).

### miR-221/222

These groups of miRNAs are highly associated with expression in vascular smooth muscle cells (VSMCs) and endothelial cells. These miRNAs have the properties of pro-migration, pro-proliferative and anti-apoptotic effects in VSMCs, whereas they exhibit anti-proliferative, anti-migration and pro-apoptotic properties in endothelial cells (103). This finding was also supported in previous research when the expression of miR-221/222 was reduced in the myocardium in severe cardiac fibrosis in patients with heart failure (104,105). However, its overexpression has been found to be responsible for causing cardiac hypertrophy *in vitro*, which leads to heart failure (106). In other studies, when mice were treated with DOX, the levels of miR-221/222 were upregulated, which was also observed following treatment with radiotherapy (107). This could be used as a basis to monitor miR-221/222 levels for diagnosis or therapeutic monitoring for cardiotoxicity.

### miR-320a

These are the group of five members of miRNAs, namely miR-3201, b, c, d and e, which regulate the apoptosis and glucose-induced gene expression in diabetes (108,109). The levels of miR-320a increase in cardiomyocytes and endothelial cells following DOX-therapy, as it targets VEGF-A; hence, when miR-320a is upregulated, there is a decrease in VEGF-A levels, altering cardiac vascular homeostasis (110). It has also been found that miR-320a can decrease DOX-medicated apoptosis. Additionally, miR-320a overexpression leads to worsening outcomes as it impairs NO release, and endothelial cell migration (110). Additionally, miR-320a targets certain molecules involved in angiogenesis, such as insulin-like growth factor and neuropilin-1(101). The levels of miR-320a have been found to be downregulated in DOX-treated subjects with acute myeloid leukemia and can be further used to elucidate its possible role in therapeutic options, while monitoring cardiotoxicity induced by DOX (110).

## 5. Clinical utility and applications

### Role of exosomal miRNAs as early warning systems for cardiotoxicity

Exosomal miRNAs serve as promising tools for the early detection of cardiotoxicity due to their unique properties, including stability in biological fluids and ability to reflect cellular stress and damage. Unlike traditional biomarkers, such as troponins, which often indicate damage following a significant myocardial injury, miRNAs can be used to detect changes earlier. Nano-sized exosomes serve as carriers for miRNAs, facilitating their transport and detection in blood, saliva, or urine. For instance, the study by Todorova *et al* (111) found that exosomal miR-208a and miR-499, which are released by cardiomyocytes, were significantly increased in patients undergoing anthracycline-based chemotherapy weeks before their LVEF began to decline. These miRNAs are specific to heart tissue, making them reliable early indicators of heart stress (111). Similarly, Totoń-Żurańska *et al* (112) found that the miRNA composition of plasma extracellular vesicles could predict late cardiotoxicity of doxorubicin, signifying their potential in early detection. By providing a non-invasive and dynamic measure of myocardial stress, exosomal miRNAs can identify patients who are at risk of developing cardiotoxicity early during their treatment course.

While the majority of existing studies focus on the diagnostic role of exosomal miRNAs following the onset of cardiac injury, a growing body of evidence supports their predictive potential, identifying cardiotoxicity before clinical or subclinical manifestations occur. For instance, Todorova *et al* (111) found that the plasma levels of exosomal miR-208a and miR-499 increased by ~2.5- and 3.2-fold, respectively, in patients with breast cancer weeks before a ≥10% decline in LVEF was observed. Similarly, Totoń-Żurańska *et al* (112) reported that plasma exosomal miR-144-3p and miR-423-3p had combined sensitivities of 78.6% and specificities of 82.1% (AUC, 0.85) for predicting cardiotoxicity in pediatric patients with leukemia receiving DOX. Furthermore, in patients treated with trastuzumab, emerging studies have identified circulating miRNAs, such as miR-148a-3p, the levels of which change early during therapy, potentially prior to troponin elevation or functional LVEF decline, suggesting predictive utility in cardiotoxicity monitoring (113). In a previous study, in a clinical cohort of patients with breast cancer treated with DOX, circulating miR-1 demonstrated an AUC of 0.851 (95% CI, 0.729-0.933), markedly outperforming cardiac troponin I (AUC, 0.544). This ROC analysis corresponded to a sensitivity of ~85% and specificity of ~80% in discriminating patients who developed cardiotoxicity vs. those who did not (114). In another prospective study by Leger *et al* (75), the baseline plasma levels of miR-499a-5p, miR-885-5p and miR-122-5p were significantly higher in patients who later developed troponin elevation during doxorubicin therapy, and their combined signature achieved an AUC of 0.79 for predicting cardiotoxicity prior to treatment initiation. However, individual sensitivity and specificity values were not reported separately, and similar data on miR-29b as a predictor at baseline are lacking (75). These findings collectively demonstrate the importance of longitudinal miRNA profiling and support the role of exosomal miRNAs as early predictive biomarkers of chemotherapy-induced cardiotoxicity. However, larger prospective trials are warranted to validate their predictive performance and integrate them into clinical workflows (115).

### Potential to guide dose adjustments or alternative treatment strategies

Exosomal miRNAs can serve as both diagnostic and therapeutic guidance. Clinicians can predict the likelihood of a patient to develop cardiotoxicity by monitoring particular miRNA patterns, and then modifying chemotherapy doses to reduce the risk. For example, patients with higher levels of miRNAs linked to cardiac stress may be switched to less cardiotoxic regimens or administered lower dosages of anthracyclines. Additionally, patients who would benefit from cardioprotective medications, such as dexrazoxane or closer monitoring using imaging and biomarkers could be identified with exosomal miRNA analysis. The previous systematic review by Brown *et al* (116) suggested that panels of miRNAs tailored to specific chemotherapy agents could guide treatment modifications to reduce cardiotoxicity in patients with breast cancer. In another study on pediatric patients with leukemia, Totoń-Żurańska *et al* (112) explored miRNAs such as miR-144-3p and miR-423-3p in patients undergoing doxorubicin treatment. These miRNAs were associated with subclinical cardiac damage, leading clinicians to modify dosages and incorporate cardioprotective agents like dexrazoxane (112). This approach significantly reduced the incidence of cardiac events. In addition to assisting with dose modifications, miRNA analysis may help guide the use of alternative medicines, enabling successful cancer treatment while lowering the risk of long-term cardiac conditions.

### Complementary use with imaging and traditional biomarkers (e.g., troponins, BNP)

The combination of exosomal miRNAs with traditional biomarkers and imaging modalities can provide a comprehensive approach to monitoring cardiotoxicity. Although biomarkers for cardiac injury and stress, such as troponins and B-type natriuretic peptide, are well-established, they are frequently discovered after substantial damage has been done. Imaging methods such as cardiac MRI and echocardiography provide structural and functional insight; however, may not always detect subclinical changes (117). By acting as early molecular indicators of cardiac stress, exosomal miRNAs may be able to close this gap. When used in conjunction with conventional techniques, miRNAs may improve the sensitivity and specificity of cardiotoxicity detection (116). A multimodal approach that includes troponins, imaging, and miRNA levels could be used to stratify patients into different categories and tailor effective treatment plans.

## 6. Technical challenges and limitations

### Variability in exosomal release in cases of carcinoma

All types of cells produce exosomes; however, their production quantity and characteristics are determined by the type of cells from which they originate. For instance, the number of exosomes produced by normal mammary epithelium is far lower than those produced by cells of breast carcinoma (118). Constituents within an exosome, such as miRNAs or microvesicles are more abundant in cells of breast carcinoma than in the normal epithelium (119). The noted difference is primarily on account of the change observed in the stromal environment. In cases of carcinoma, it is driven by transforming growth factor-D, functionally active membrane type 1-matrix metalloprotease) (120). Other common extrinsic factors influencing stromal environments include chemical compounds such as cannabidiol. It is known to inhibit exosomal and microvesicle release in certain types of cancer, such as breast adenocarcinoma (MDA-MB-231), prostate cancer (PC3) and hepatocellular carcinoma (HEPG2) cells. Its effect is observed in a concentration-dependent manner. Cannabidiol has been explored as an anticancer agent due to its ability to inhibit exosome release (121). A similar role is also demonstrated by sulfisoxazole, particularly in cases of breast carcinoma. It functions by inhibiting small extracellular vesicles. The inhibition of small extracellular vesicles leads to the degradation of exosomes through lysosomal action (122). Another factor that results in the quantum of exosomes being released is the involvement of adipocytes or the proximity of the tumor cells to adipose tissue. The activation of adipocytes by tumor cells leads to the secretion of MFG-E8-associated exosomes, which further transfer necessary RNAs required for lipid synthesis. Lipids form the primary energy source for these tumor cells thus promoting spread and local invasion (123,124). Thus, in tumorogenesis, there exists a positive feedback mechanism driving exosomal production.

### Exosomal isolation and miRNA quantification: Challenges and current techniques

For the isolation of exosomes and miRNAs, there exists no standardized protocol. The most widely used methods include differential centrifugation and ultracentrifugation. Challenges accounted with these methods include damage to exosomes, and reduced final yields leading to low miRNA, and RNA yields. Other issues include co-isolation of non-exosomal impurities, and low reproducibility. Together, these drawbacks lead to the isolation of samples that are incompatible to be used for clinical purposes (125). For isolation in media comprising biological fluids, such as urine, blood, or plasma, a combination of methods is used. The most prominent of these is low-speed density gradient centrifugation. This process removes large platelet-derived vesicles and numerous other cellular fragments. Followoing this, the sample is further processed through ultrafilteration and size exclusion chromatography (126). The desired outcome of any extraction process remains near 100% exosomal purity devoid of any contaminants this goal though is unrealistic. Size exclusion chromatography is one of the methods which result in the good extraction of pure exosomes from samples with the least amount of contamination from the currently available methods (127). Another promising technique is the asymmetric-flow field-flow fractionation. It employs crossflows and variable forward laminar flow as a means to segregate nanoparticles based on their respective densities and unique hydrodynamic qualities. This technique further leads to the identification of various subpopulations within exosomes and exomers (128). Ultimately each method has its unique pros and cons and may be suitable for extraction in certain specific conditions and tissue samples.

### Barriers to the clinical translation of exosomal miRNA biomarkers

In addition to technical limitations related to exosomal isolation and miRNA quantification, several broader challenges impede the clinical implementation of exosomal miRNA biomarkers. There is currently no global consensus on standardized protocols for exosome purification, miRNA normalization, and data reporting resulting in considerable inter-laboratory variability and poor reproducibility across studies (129). Secondly, demographic characteristics such as age, sex, ethnicity and baseline cardiovascular risk factors have been shown to influence circulating miRNA profiles, introducing potential variability that must be carefully controlled in clinical validation studies (130). Additionally, comorbid conditions, including hypertension, diabetes mellitus, chronic kidney disease, and systemic inflammation, independently modulate miRNA expression and can confound their specificity as biomarkers of chemotherapy-induced cardiotoxicity (131,132). Furthermore, dataset shifts such as reduced performance when applying models developed in one population to a different one highlight the need for multicenter, ethnically diverse prospective trials to validate cut-off values and biomarker performance across heterogeneous populations (133). Finally, logistical considerations, such as cost-effectiveness, turnaround time, and integration into existing clinical workflows, remain largely unaddressed and pose practical barriers to routine implementation in cardio-oncology care pathways (134).

## 7. Future directions

### Development of multiplex panels combining exosomal miRNAs with other liquid biopsy markers

Developing reliable multiplex panels that combine exosomal miRNAs with other indicators, including circulating tumor DNA, proteins and metabolites, is the key to predicting chemotherapy-induced cardiotoxicity in the future. By improving sensitivity and specificity, these panels could offer a thorough picture of the patient's physiological and biochemical reaction to therapy. For instance, early identification and prognostication may be enhanced by combining miRNA profiles with cardiac-specific biomarkers such as troponin or NT-proBNP (135). Such multiplex techniques will also help differentiate chemotherapy-induced cardiotoxicity from other cardiac diseases, providing more targeted interventions.

### Integration with AI and machine learning for predictive modeling

Exosomal miRNA datasets combined with AI and machine learning techniques can potentially transform predictive modeling for cardiotoxicity completely. AI algorithms can recognize intricate patterns in miRNA expression profiles, which makes it easier to create prediction risk assessment scores. Machine learning models, such as random forests and neural networks, can adaptively learn from massive datasets to improve predictions over time to accommodate patient heterogeneity (136). This method may open the door for real-time clinical decision support systems that use patient-specific information to categorize risk and provide individualized treatment plans.

### Role of miRNAs as medical intervention drugs

miRNA-based treatments, which target gene expression pathways, are exhibiting promise as a means of treating complicated disorders. Therapeutic strategies include antagomirs to block overexpressed miRNAs and mimic to restore underexpressed ones. In disorders including cancer, heart disease and chemotherapy-induced cardiotoxicity, these methods target molecular dysregulations: i) MRX34: A miR-34 mimic created for cancer treatment, it demonstrated promise by regaining its ability to control tumor growth. However, immune-related adverse events led to the termination of its clinical trial, revealing issues with delivery and safety (137). ii) SPC3649 (miravirsen): This antagomir targets miR-122, demonstrating efficacy in reducing hepatitis C virus replication and achieving success in Phase II trials (137,138). iii) CDR132L: miR-132 is the target of this antimiR, which leads to unfavorable heart remodeling. It has been proposed as a possible treatment for heart failure after early-phase clinical trials showed notable improvements in cardiac function (137-139).

## 8. Conclusion

Through the advancements that have been made in understanding the role of miRNAs and their potential application in the diagnosis of cardiotoxicity, it is relevant that they may serve as biomarkers in the future. One of the limitations in currently available data may be that a number of miRNAs that have been reported to be linked with cardiotoxicity may have been due to the limited research and could thus act as a bias. Further studies that could replicate the results of certain miRNAs in cardiotoxicity are warranted. The present review demonstrates that miRNAs may be used informative biomarkers, providing further more evidence from clinical studies on miRNAs in cardiovascular disease. Apart from certain miRNAs examined as biomarkers to evaluate cardiotoxicity in cancer care, miRNAs have also been explored in therapeutic approaches to deliver drugs or modify the signaling pathways. To support the collected evidence, farther studies are required to assess the potential of miRNAs in cardiotoxicity due to chemotherapeutic agents.

## Figures and Tables

**Table I:** Various exosomal miRNAs associated with certain pathologies.

Exosomal mmiRNAs	Responsible for the pathology	(Refs.)
miR-1246 and miR-4644	Salivary exosomes were significantly higher in cancer patients with a potential for use in the diagnosis of pancreaticobiliary tract cancer.	(140)
bta-miR-142-5p and bta-miR-223	Significantly upregulated in bovine milk in response to bacterial infection of intramammary glands providing a potential to be used for early detection of bacterial infections in mammary glands.	(141)
Exosomal RNAs from amniotic fluids	Used for CD24 Ala/Val SNP genotyping responsible for the more rapid progression of autoimmune diseases.	(42)

**Table II:** Key exosomal miRNAs and their roles in chemotherapy-induced cardiotoxicity.

miRNA Name	Role in cardiotoxicity	Key mechanisms/findings	(Refs.)
miRNA-200c	Endothelial dysfunction and apoptosis	Upregulated by DOXO, decreases ZEB1, SIRT1, and FOXO1; causes oxidative stress-induced damage.	(51,53)
miRNA-200a	Protective against oxidative stress	Targets Keap1, activates Nrf2; reduced levels by DOXO increase apoptosis.	(62)
miR-34a	Apoptosis and ROS generation	Targets Bcl-2, activates caspase-3, affects SIRT1/p66shc; reversible with Dexrazoxane.	(58)
miR-34b/c	Inflammatory response	Targets ITCH protein; increases TNF-α and IL-6 levels.	(56)
miR-29a	Cardiac remodeling	Upregulated post-myocardial injury; correlates with fibrosis.	(72,73)
miR-29b	Anti-apoptotic role	Downregulated by DOXO; targets Bax to prevent myocyte damage.	(74)
miR-30a-e	Cardioprotective role	Downregulated by DOXO; linked to GATA6 inhibition and cardiotoxicity.	(81)
miR-21	Dual role: protective and damaging	Promotes anti-apoptotic pathways via programmed cell death 4 (PDCD4) inhibition but chronic overexpression impairs cardiac function.	(84,85)

DOXO, doxorubicin; ZEB1, zinc finger E-box binding homeobox 1; SIRT1, sirtuin 1; FOXO1, forkhead box O1; ROS, reactive oxygen species; GATA6, GATA binding protein 6.

## Data Availability

Not applicable.
